# Novel Insights into the Potential Diagnostic Value of Circulating Exosomal IncRNA-Related Networks in Large Artery Atherosclerotic Stroke

**DOI:** 10.3389/fmolb.2021.682769

**Published:** 2021-05-21

**Authors:** Shuai Zhang, Jing Wang, Mei Jie Qu, Kun Wang, Ai Jun Ma, Xu Dong Pan, Xiao Yan Zhu

**Affiliations:** ^1^ Department of Neurology, The Affiliated Hospital of Qingdao University, Qingdao, China; ^2^ Institute of Cerebrovascular Diseases, The Affiliated Hospital of Qingdao University, Qingdao, China; ^3^ Department of Critical Care Medicine, The Affiliated Hospital of Qingdao University, Qingdao, China

**Keywords:** large artery atherosclerotic stroke, exosome, long noncoding RNA, diagnostic performance, RNA sequencing

## Abstract

Exosomes show diagnostic and therapeutic promise as carriers of ncRNAs in diseases. LncRNAs in exosomes have been identified as being stable and avoided degradation by nucleolytic enzymes. Although lncRNAs have been confirmed to be important in cancers, no studies for exo-lncRNAs have been reported in LAA stroke. High-throughput sequencing was performed to detect the differential expression profiles of lncRNAs in five paired plasma-derived exosome samples from patients with LAA stroke and controls (matched on vascular risk factors). Exo-lncRNA-associated networks were predicted with a combination of multiple databases. The expression of the selected genes in the networks was confirmed by qRT-PCR in a validation set (LAA vs. controls = 30:30). Furthermore, ROC analysis was used to evaluate the diagnostic performance of the lncRNA-related networks. A total of 1,020 differentially expressed lncRNAs were identified in LAA stroke patients. GO and KEGG pathway analyses indicated that their target genes are involved in atherosclerosis-related pathways, including inflammation, cell adhesion, and cell migration. qRT-PCR confirmed that the expression trend of differential expressed genes was consistent with RNA-seq. Furthermore, the AUCs of the lnc_002015-related network and lnc_001350-related network were 0.959 and 0.97, respectively, in LAA stroke. Our study showed the differential expression of lncRNAs in plasma exosomes and presented related diagnostic networks for LAA stroke for the first time. The results suggested that exosomal lncRNA-related networks could be potential diagnostic tools in LAA stroke.

## Introduction

Stroke, the second leading cause of global mortality (Chen et al., 2017; Zhang et al., 2020), is also widely known for its high disability rate. Large artery atherosclerotic (LAA) stroke, classified according to the Trial of Org 10,172 in Acute Stroke Treatment (TOAST) system, is the most common type of stroke (Adams et al., 1993; Huang et al., 2017). LAA stroke has the highest rates of recurrence and death among all stroke subtypes (Murat Sumer et al., 2002). Thrombolytic therapy plays an important role in the treatment of ischemic stroke, but it has a strict time window (Lansberg et al., 2009; Sidorov et al., 2019). Thus, a rapid and accurate blood test for LAA stroke would be valuable. Increasing studies have verified that exosomes, as a form of communication between cells, have promise for diagnosis and treatment in cancer and neurodegenerative diseases (Zheng et al., 2019; Khalyfa et al., 2020; Jia et al., 2021).

Exosomes, 30–150 nm in size, are formed by the invagination of intracellular lysosome particles and their fusion with the cell membrane (Al-Nedawi et al., 2008). When exosomes are incorporated into recipient cells, their contents, such as RNA molecules (e.g., lncRNAs, circRNAs, miRNAs, mRNAs), proteins and lipids, are released into recipient cells, thus acting as a type of cell-cell communication (Al-Nedawi et al., 2008; Jeppesen et al., 2019). These different cargos make exosomes to be unique candidate of noninvasive diagnostic indicators. Evidence has shown that lncRNA can be protected from degradation by exosomes, which increases the stability of lncRNAs in plasma (Guo et al., 2020; Kim et al., 2020). A previous study verified that in hepatocellular carcinoma, circulating lncRNA-GC1 molecules were almost all packaged in exosomes and thereby protected, ensuring sufficient stability of lncRNA-GC1 in clinical tests (Lu et al., 2020). Thus, exosomes could be potential targets for exploring the diagnostic and regulatory mechanisms of lncRNAs.

LncRNAs, which are transcribed sequences longer than 200 nucleotides, have been recognized and proven to be important participants in biological functions, such as inflammation, immunity and autophagy (Serghiou et al., 2016; Robinson et al., 2020). LncRNAs have a variety of regulatory functions, and the ceRNA network regulatory mechanism is one of the most important posttranscriptional regulation mechanisms in the cytoplasm (Cesana et al., 2011). Existing studies have found that lncRNAs can competitively bind miRNAs, thus influencing the regulation of downstream target mRNAs by miRNAs (Qu et al., 2016; Huang et al., 2019). Recently, Dykstra-Aiello et al. suggested that lncRNAs might be included with miRNAs and mRNAs as possible diagnostic indicators in stroke (Dykstra-Aiello et al., 2016). However, no studies have assessed the potential diagnostic value of exo-lncRNA-related networks.

In general, this study aimed to explore the differential expression profiles of exo-lncRNAs and construct exo-lncRNA-related networks, which might be evaluated for their potential utility in the accurate diagnosis of LAA stroke. Our results may provide a novel perspective on exo-lncRNA-related networks for the diagnosis and understanding of the mechanism of LAA stroke.

## Materials and Methods

### Study Population Enrollment

Our study enrolled 35 LAA stroke patients (LAA group) and 35 healthy persons (control group) at the Affiliated Hospital of Qingdao University from March 2020–October 2020. The discovery samples included 5 patients and 5 healthy controls. Validation was performed in 30 patients and 30 healthy controls. All samples were matched on age, sex, and vascular risk factors (VRFs) including hypertension, diabetes mellitus, smoking and drinking.

LAA stroke patients (LAA group) were included in the study according to the TOAST standards (Adams et al., 1993). Inclusion criteria were as follows: ischemic stroke within 72 h of onset; age ≥ 18 years; underwent brain MRI, brain MRA or CTA examination and echocardiography; and the stenosis rate of extracranial or intracranial arteries was more than 50%. The correlation between the degree of atherosclerosis and stroke was determined by experienced neuroscientists. Exclusion criteria included: nonatherosclerotic intracranial stenosis (e.g., moyamoya disease, vasculitis, or anatomy), potential sources of cardiac embolism (e.g., atrial fibrillation), incomplete cerebrovascular condition examination, and tumor or severe liver and kidney dysfunction (Feng et al., 2019; Shen et al., 2019). This study was approved by the Ethics Committee of The Affiliated Hospital of Qingdao University.

### Blood Collection Exosome Isolation

Peripheral blood samples (6 ml) were collected in EDTA tubes. After centrifugation at 3,000 × *g* at 4°C for 15 min, at least 3 ml plasma was collected and stored at −80°C. After thawing at 37°C, the plasma was centrifuged at 2,000 × g for 20 min, and the supernatant was obtained. The debris was centrifuged again at 10,000 × g for 20 min. Then, Total Exosome Isolation (from plasma) reagent (Invitrogen, 4484450, California, United States) (Lobb, et al., 2015) was used according to the manufacturer’s instructions. Briefly, protease K (0.05 times the volume of plasma) was added to the plasma and incubated at 37°C for 10 min. Then, 0.2 volumes of exosome extraction reagent were added to the mixture and incubated at 4°C for 30 min. The mixture was centrifuged at 10,000 × g at room temperature for 5 min. The exosomes, contained in a pellet at the bottom of the tube, were resuspended in PBS and stored at −80°C.

### Identification of Plasma Exosomes

#### Identification of Exosomes by Nanoparticle Tracking Analysis

Exosome samples were resuspended in 1 ml of 1 × PBS and examined using a Zeta View PMX 110 (Particle Metrix, Meerbusch, Germany) equipped with a 405 nm laser to determine the size and quantity of particles (Zhan et al., 2018). Particle movement was assessed by NTA software (ZetaView 8.02.28).

#### Observation of Exosomes by Transmission Electron Microscopy

Exosome suspension (20 µL) was deposited on a copper grid for 10 min at room temperature, and then washed with sterile distilled water. The exosomes were negatively stained with 2% uranyl acetate solution for 1 min and dried for a few minutes at room temperature. The samples were observed and photographed under a transmission electron microscopy (Zhan et al., 2018) (TEM, Hitachi, Tokyo, Japan).

#### Analysis of Exosomes by Western Blot

Exosomes were lyzed in cold standard RIPA buffer with PMSF (MCE, United States). The total protein concentration was determined by BCA assay. Protein samples (25 ug/lane) were added to 10% SDS polyacrylamide gels and then transferred to PVDF membranes (Millipore Co., NJ, United States of America). Plasma exosomes were identified using three positive markers, CD9, CD63, and TSG101 (Abcam, MA, United States), and one negative marker, GRP94 (Abcam, MA, United States) (Cordonnier et al., 2020). The blots were detected by a chemiluminescence system (United States) and analyzed by Quantity One.

#### RNA Extraction and Quality Assessment

Total exosomal RNA was isolated by the miRNeasy Serum/Plasma Advanced Kit (Qiagen, cat. 217,204, Germany); specific procedures were performed according to the company’s operation manual. RNA purity was checked using a NanoPhotometer^®^ spectrophotometer (IMPLEN, CA, United States). RNA concentration was measured using a Qubit^®^ RNA Assay Kit in a Qubit^®^ 2.0 Fluorometer (Life Technologies, CA, United States) (Qin et al., 2018). RNA integrity was assessed using the RNA Nano 6000 Assay Kit of the Bioanalyzer 2,100 system (Agilent Technologies, CA, United States).

#### Library Construction and Sequencing

The libraries were constructed and sequenced by Novogene (Beijing, China) (Tian et al., 2020). Briefly, a total amount of 20 ng RNA per sample was used as the RNA sample for preparation. Ribosomal RNA was removed by an Epicenter Ribo-zero™ rRNA Removal Kit (Epicenter, United States). The sequencing libraries were generated using rRNA-depleted RNA by the NEBNext^®^ Ultra™ Directional RNA Library Prep Kit for Illumina^®^ (NEB, United States), following the manufacturer’s recommendations. Briefly, first strand cDNA was synthesized using random hexamer primers and M-MuLV Reverse Transcriptase (RNaseH). Second-strand cDNA synthesis was subsequently performed using DNA Polymerase I and RNase H. Then, PCR was performed with Phusion High-Fidelity DNA polymerase, universal PCR primers and Index (X) Primer. Finally, the products were purified (AMPure XP system), and the library quality was assessed on the Agilent Bioanalyzer 2,100 system. The libraries were sequenced on an Illumina HiSeq 2,500 platform, and 125 bp paired-end reads were generated.

#### Analysis and Screening of lncRNA, miRNA and mRNA Data

Raw data (raw reads) in FASTQ format were first processed through in-house Perl scripts to obtain clean data (clean reads). At the same time, the Q20, Q30, and GC contents of the clean data were calculated. All downstream analyses were based on clean data with high quality. We obtained eligible transcripts through matching, assembly, screening and evaluation. String Tie (v2.1.1) was used to calculate the FPKMs of both lncRNAs and coding transcripts in each sample.

The reads obtained from the above steps were assessed using the Ballgown suite, Cufflinks and edgeR. Transcripts with |log_2_fold change| (logFC) ≥2 (lncRNA, mRNA) or 1 (miRNA) and a *p* < 0.05 were assigned as differentially expressed. The results were visualized by volcano maps and heat maps prepared using the R package (R-3.3.3) and TBtools.

#### PPI Network and Module Analysis of DE mRNAs

PPI analysis of the differential expressed (DE) mRNAs was performed based on the STRING database. Then, the submodules were selected from the PPI network with a cutoff score = 2.5 by the Molecular Complex Detection (MCODE) plugin. The results were visualized by Cytoscape (Cytoscape-3.7.2).

#### GO and KEGG Enrichment Analysis

The DAVID database and KOBAS database were used to infer the potential functions of the DE mRNAs in terms of biological process (BP), cellular component (CC), and molecular function (MF) GO annotations and KEGG pathways. R package tools were used to visualize the results.

#### Prediction of Subcellular Localization of lncRNAs

The regulatory pattern of lncRNAs is largely dependent on their subcellular localization. The lncLocator website and iLoc-LncRNA website are useful tools for obtaining predictions of subcellular localization. A high prediction score indicates that the searched lncRNA localizes mostly to the cytoplasm. The exo-lncRNAs of interest (selection criteria according to the fold change, function of differential expressed genes and reads in each sample, the details of the data were shown in Online Supplementary Tables S1, S4, S5) were predicted, which further confirmed the lncRNA-related molecular interaction sites.

#### Construction of lncRNA-related Networks

We used the R “cor.test” package to calculate the correlation between DE lncRNAs and DE mRNAs, based on the expression levels of the genes. A correlation coefficient (Spearman correlation analysis) > 0.85 and a *p* < 0.05 were considered to indicate coexpression. LncRNAs can act as sponges by binding with the “seed” region of miRNAs. Based on TargetScan, RNAhybrid and miRanda analyses, the binding sites of miRNA- DE lncRNAs and miRNA-DE mRNAs were predicted. The same miRNA binding sites were selected. The parameters used in miRanda were S > 150 and ΔG < −30 kcal/mol. S represents the number of matching bases, and ΔG is the binding free energy of the paired sequence. The correlation coefficient between the miRNA and lncRNA or mRNA was calculated, and pairs with negative correlations were selected. The lncRNAs of interest were screened based on the target gene functions and differential expression to identify hub lncRNAs. Then, lncRNA-related networks were established and visualized via Cytoscape.

#### Validation of RNA-seq Results by Quantitative Real-Time Polymerase Chain Reaction

qRT-PCR was used to verify the DE hub lncRNAs, mRNAs and miRNAs in ceRNA networks. Plasma exosomes and exosomal RNA were extracted as previously described. cDNAs of RNAs (miRNAs, lncRNAs and mRNAs) were synthesized using a Mir-X miRNA First-Strand Synthesis Kit (Takara, 638315, Japan) and PrimeScript™ RT Kit with gDNA Eraser (Takara, RR047A, Japan), respectively. Then, gene expression levels were detected by SYBR Premix Ex Taq II (RR820A) and LightCycler 480 II systems. The primers used for qRT-PCR are shown in Table 1. ACTB and U6 were used as internal reference. The relative expression levels were calculated using the 2^−∆∆Ct^ method, where ∆Ct = Ct_target_–Ct_reference_, ∆∆Ct = ∆Ct _LAA_–∆Ct_control_.

**TABLE 1:** Primer sequences in the study.

Genes	5′–3′	Primer sequences
lnc_000048	Forward	TGG​GCG​GGA​TTC​TGA​CTT​AGA​GG
	Reverse	GGT​GTA​TGT​GCT​TGG​CTG​AGG​AG
lnc_001346	Forward	TGG​TAG​AGT​AGA​TGA​CGG​GTT​GGG
	Reverse	AAA​CTT​CCT​GCC​ACC​TAT​CAC​ACC
lnc_001347	Forward	TGG​GTT​CGA​TTC​TCA​TAG​TCC​TAG​A
	Reverse	CCA​TAC​CCA​TTA​CAA​TCT​CCA​GCA
lnc_001350	Forward	CCA​CCT​CAA​CTG​CCT​GCC​ATG
	Reverse	ACA​TCC​GGC​CTG​CTC​CTT​CTC
lnc_002015	Forward	GCG​TTC​AGC​ACC​ATC​ACT​TCT​TTG
	Reverse	CCT​CCA​TCA​ACA​CCA​AGC​AGC​AG
lnc_012006	Forward	TCC​TTC​TTC​CAG​TGC​TCC​TCT​TCC
	Reverse	TGG​TTC​CTC​TTG​GGT​CCT​TCC​G
lnc_013144	Forward	TGA​ATA​CAG​CCA​GCT​CTC​CTC​CTC
	Reverse	AGG​CGG​ACG​AGT​AGC​GAA​GAG
lnc_016442	Forward	CCT​CTG​TCT​GTC​TCT​GTC​CCT​CTC
	Reverse	CCC​GAC​CAG​GAT​GCC​AGG​AG
AACS	Forward	GGC​ACC​CTC​ATC​CAG​CAT​CT
	Reverse	TCC​ACA​TCA​TCC​AGC​CGA​CC
AAMP	Forward	CCT​GCC​TGT​GTG​TCT​GGT​GTT​G
	Reverse	CTC​CTC​TTC​CCT​CTG​TGC​CCT​AC
ABCA3	Forward	CCG​GGA​GAT​GCT​GGT​CAT​GT
	Reverse	TCC​TGA​CCA​GCT​TGT​TGG​CA
GADD45G	Forward	GCG​TCT​ACG​AGT​CAG​CCA​AAG​TC
	Reverse	GTC​GCC​CTC​GTC​CTC​CTC​AC
JUNB	Forward	CAC​GAC​GAC​TCA​TAC​ACA​GCT​ACG
	Reverse	CCA​GGC​TCG​GTT​TCA​GGA​GTT​TG
PTPRC	Forward	AGA​CGC​CCT​CTG​CTG​GAA​CTG
	Reverse	GCT​GGC​TGT​ACT​CCT​CTC​TCC​TG
ST14	Forward	CAC​ACC​TAC​CGC​TGC​CTC​AAT​G
	Reverse	CCG​TCG​CTA​CAG​TCC​TCC​TTC​C
ACTB	Forward	GCG​GAC​TAT​GAC​TTA​GTT​GCG​TTA​CA
	Reverse	TGC​TGT​CAC​CTT​CAC​CGT​TCC​A
U6	Forward	CTCGCTTCGGCAGCACA
	Reverse	AAC​GCT​TCA​CGA​ATT​TGC​GT
Hsa-miR-328	Forward	CTGGCCCTCTCTGCCCTT
Hsa-miR-342	Forward	TCT​CAC​ACA​GAA​ATC​GCA​CCC​G
Hsa-miR-1273	Forward	CTG​CAG​ACT​CGA​CCT​CCC​AG
Hsa-miR-1538	Forward	TAT​ATA​CGG​CCC​GGG​CTG​C
Hsa-miR-1908	Forward	TAT​ATA​CGG​CGG​GGA​CGG​C
Hsa-miR-3127	Forward	TCC​CCT​TCT​GCA​GGC​CTG​CGG

#### Statistical Analysis

Statistical analysis was performed using SPSS (SPSS 13.0) and MedCalc software. The data are presented as the mean ± standard deviation (SD) or the medians (interquartile ranges). Independent-samples t-tests, Chi-square tests and Wilcoxon rank sum tests were used to analyze the differences between 2 groups. Logistic regression analysis was used to identify the risk factors for LAA ischemic stroke and generate the diagnostic panel. Spearman correlation analysis was used to detect relationships between RNAs. A receiver operating characteristic (ROC) curve was generated, and the area under the curve (AUC) was calculated to evaluate the diagnostic performance of differentially expressed RNAs, diagnostic panels, and diagnostic networks. *p* < 0.05 were considered to be statistically significant. R packages, TB Tools, and MedCalc were used for visualization of the results.

#### Data Availability Statement

Raw data files have been deposited to the Gene Expression Omnibus Database (www.ncbi.nlm.nih.gov/geo/) and accession number is GSE173719.

## Results

### Basic Characteristics of the Participant Population

The basic characteristics of 35 LAA stroke patients and 35 healthy persons were shown in Table 2. There were no significant differences in sex, age, hypertension, diabetes, smoking, alcohol consumption, TG, TC, LDL, and HDL between the two groups (*p* > 0.05).

**TABLE 2:** Baseline characteristics of the subjects.

Variables	Control group	LAA group	*p* value
Sample size (%)	35	35	—
Gender, number (%)			
Female	16 (45.7)	14 (40)	0.629^a^
Male	19 (54.3)	21 (60)	
Age (years), mean ± SD	62.54 ± 9.21	65.40 ± 9.31	0.201^b^
BMI (kg/m^2^), media (IQR)	22.86 (1.67)	22.15 (2.33)	0.051^b^
Diabetes, number (%)			
Yes	16 (45.7)	14 (40)	0.629^a^
No	19 (54.3)	21 (60)	
Hypertension, number (%)			
Yes	17 (48.6)	19 (54.3)	0.632^a^
No	18 (51.4)	16 (45.7)	
Smoke, number (%)			
Yes	18 (51.4)	20 (57.1)	0.631^a^
No	17 (48.6)	15 (42.9)	
Alcohol, number (%)			
Yes	16 (45.7)	15 (42.9)	0.81^a^
No	19 (54.3)	20 (57.1)	
TC	1.21 ± 0.65	1.41 ± 0.75	0.299^b^
TG	4.13 ± 1.02	4.30 ± 0.83	0.299^b^
LDL	2.53 ± 0.89	2.26 ± 0.64	0.719^b^
HDL	1.19 ± 0.47	1.17 ± 0.34	0.977^b^

a Chi-square tests.

b Two independent sample t tests.

*p* < 0.05 was considered to indicate statistical significance.

### Identification of Plasma-Derived Exosomes

**FIGURE 1:**
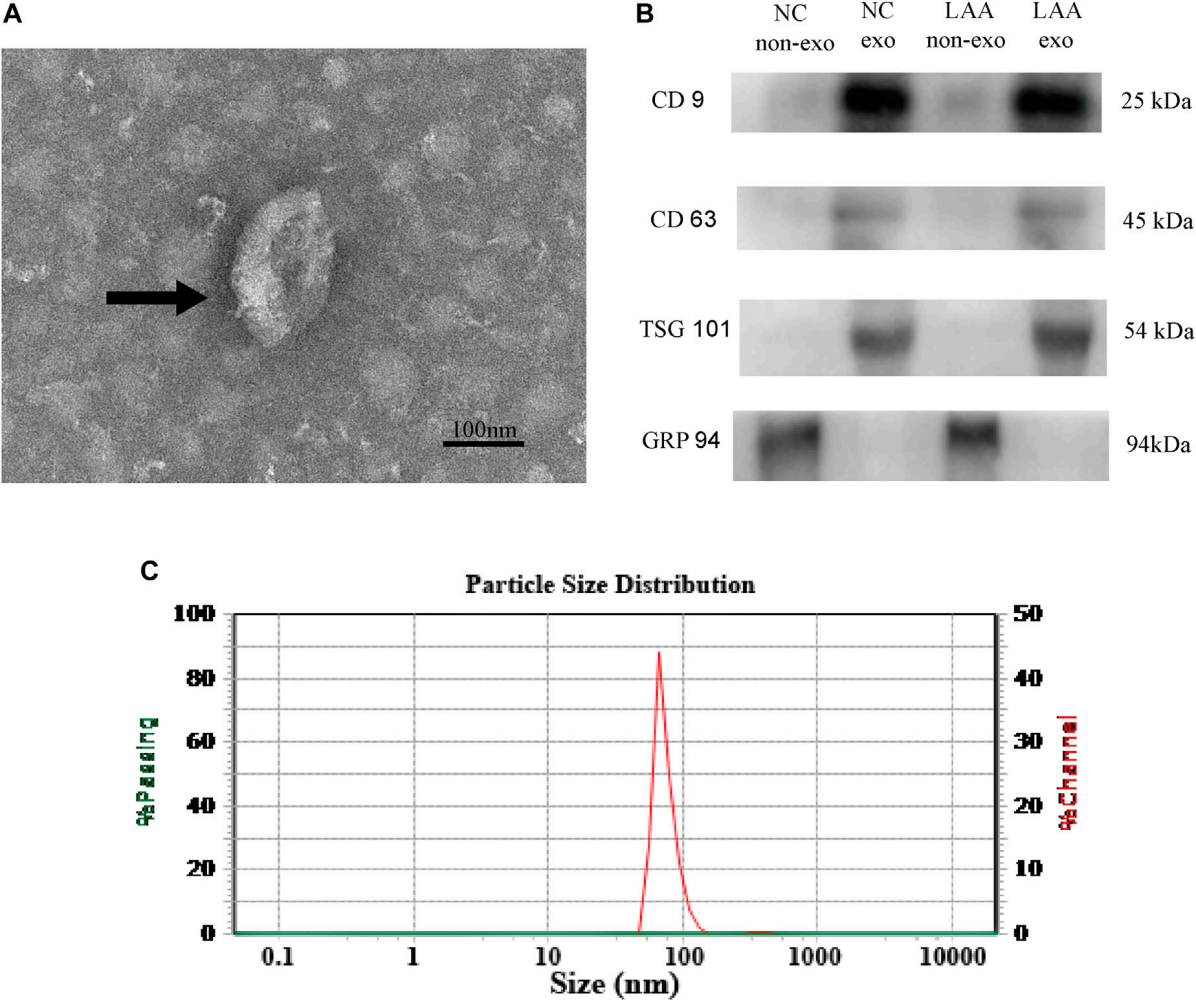
Characteristics of plasma-derived exosomes. **(A)** Plasma-derived exosomes were analyzed under TEM and exhibited a cup-shaped membrane morphology with a diameter of 30–150 nm. Typical exosomes were indicated using black arrows. Figure scale bar = 100 nm. **(B)** Exosome-enriched protein markers, including CD9 (25 kDa) and TSG101 (54 kDa) and CD63 (45 kDa), and a negative marker, GRP94 (94 kDa), were analyzed by Western blotting in exosomes. Lane 1: plasma without exosome of control; lane 2: plasma with exosome of control; lane 3: plasma without exosome of LAA; lane 4: plasma with exosome of LAA. **(C)** NTA confirmed that the size of the exosomes mainly ranged from 30–150 nm in diameter.

Plasma-derived exosomes were analyzed and verified by electron microscopy, nanoparticle tracking analysis and Western blot. The presence of exosomes was confirmed by TEM, which detected cup-shaped bilayer vesicles (Figure 1A). Western blotting analysis was used to estimate the exosome purity. Three positive protein markers (CD9, CD63, and TSG101) of exosomes were all detected; conversely, GRP 94, a negative marker of exosomes, was absent from the isolated exosomes (Figure 1B). Nanoparticle tracking analysis demonstrated that the size distribution of the exosomes mainly ranged from 30–150 nm, accounting for 98.72% of the total particle number (Figure 1C). The TEM, NTA, and Western blot results were consistent with the characteristics of exosomes.

### Differential Expression Analysis

**FIGURE 2:**
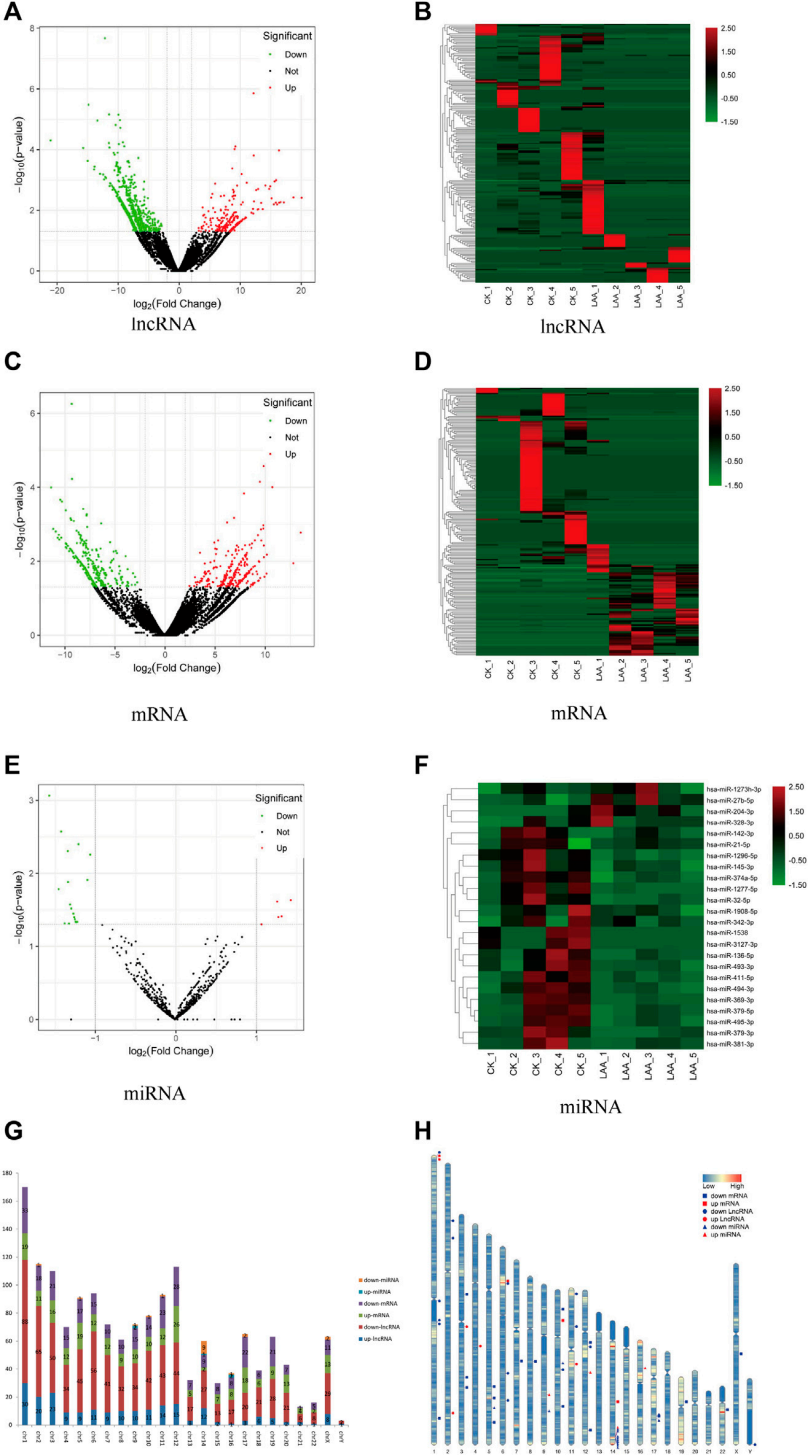
Analysis of differently expressed RNAs. Significantly changed lncRNAs **(A)**, mRNAs **(C)**, and miRNAs **(E)** were visualized in volcano plots. Cluster heatmap of differentially expressed lncRNAs **(B)**, mRNAs **(D)** and miRNAs **(F)**. Red represents upregulated genes, and green represents downregulated genes. **(G)** The distributions of the differentially expressed RNAs on all 23 chromosomes. Red is the number of downregulated lncRNAs, blue is the number of upregulated lncRNA, orange is the number of downregulated miRNAs, light blue is the number of upregulated miRNAs, purple is the number of downregulated mRNAs and green is the number of upregulated mRNAs. **(H)** Location of TOP 20 DE lncRNAs, DE mRNAs and miRNAs on every chromosome. Red represents upregulated genes, blue represents downregulated genes, circle represents lncRNA, box represents mRNA and triangle represents miRNA.

A total of 1,020 lncRNAs were differentially expressed in the LAA group compared to the control group (Figures 2A,B), of which 226 were significantly upregulated and 794 were significantly downregulated. A total of 579 differentially expressed mRNA transcripts (Figures 2C,D) were detected, of which 255 were upregulated and 324 were downregulated. A total of 24 differentially expressed miRNAs were detected (Figures 2E,F), of which 5 were upregulated and 19 were downregulated. For lncRNAs, we screened new, previously unstudied lncRNAs for subsequent analysis. As presented in Table 3, the most upregulated lncRNA, mRNA, and miRNA were novel lnc_000288 (logFC = 20.25), FTH1 (logFC = 13.66), and hsa-miR-27 (logFC = 1.32), respectively. The most downregulated lncRNA, miRNA, and mRNA were novel lnc_000285 (logFC = −20.87), ELF1 (logFC = −11.27), and hsa-miR-1277 (logFC = -1.56), respectively (Table 3, Online Supplementary Tables S1–S3).

**TABLE 3:** Top 10 exo-lncRNA, exo-miRNA, exo-mRNA respectively ranked by logFC (RNA-seq data).

transcript_id	Gene_Type	Chr	Length	logFC	*p* value
LNC_000288	LincRNA	chr11	889	20.25	0.00387
LNC_000287	lincRNA	chr3	388	18.91	0.00375
LNC_011780	antisense_lncRNA	chr3	855	17.35	0.00548
LNC_000291	lincRNA	chr8	316	16.81	0.00539
LNC_000304	lincRNA	chr3	638	16.56	0.00559
LNC_000285	lincRNA	chr11	558	−20.87	4.95E-05
LNC_011782	antisense_lncRNA	chr3	938	−15.53	8.79E-05
LNC_001060	intronic_lncRNA	chr1	1,312	−14.65	3.33E-06
LNC_001349	lincRNA	chr1	1,151	−13.75	0.00044
LNC_000047	lincRNA	chr9	1,151	−13.68	0.000363
FTH1	protein_coding	chr11	786	13.66	0.00167
MAP3K7CL	protein_coding	chr21	1904	12.92	0.01124
CTTN	protein_coding	chr11	3,249	10.83	9.92E-05
FYB1	protein_coding	chr5	4,731	10.28	0.006626
ARPC1B	protein_coding	chr7	1,509	10.24	0.021709
ELF1	protein_coding	chr13	2,116	−11.27	0.00010
YWHAZ	protein_coding	chr8	3,007	−11.07	0.00133
HIPK3	protein_coding	chr11	7,345	−10.81	0.00157
SEPTIN7	protein_coding	chr7	4,350	−10.62	0.00231
ASAH1	protein_coding	chr8	1724	−10.51	0.00194
Hsa-miR-27 b	microRNA	chr9	96	1.32	0.03870
Hsa-miR-328	microRNA	chr 16	74	1.32	0.02333
Hsa-miR-1273 h	microRNA	chr 16	115	1.29	0.03974
Hsa-miR-204	microRNA	chr 9	109	1.27	0.02428
Hsa-miR-342	microRNA	chr 14	98	1.075	0.04988
Hsa-miR-1277	microRNA	chr x	77	−1.56	0.00087
Hsa-miR-1538	microRNA	chr 16	60	−1.44	0.01645
Hsa-miR-369	microRNA	chr 14	69	−1.41	0.00267
Hsa-miR-21	MicroRNA	chr 17	71	−1.37	0.04814
Hsa-miR-1296	MicroRNA	chr 10	91	−1.33	0.00496

We determined the chromosomal distribution of the differentially expressed genes (Figure 2G). The map shows that each chromosome contains differentially expressed RNAs, and chromosome 1 has the most differentiated transcripts. Genes located closer to one another on chromosomes may perform similar biological functions or participate in the same metabolic pathways. We therefore also visualized the distribution of DE RNAs within each chromosome (Figure 2H).

### Functional Enrichment Analysis of Differential mRNA

**FIGURE 3:**
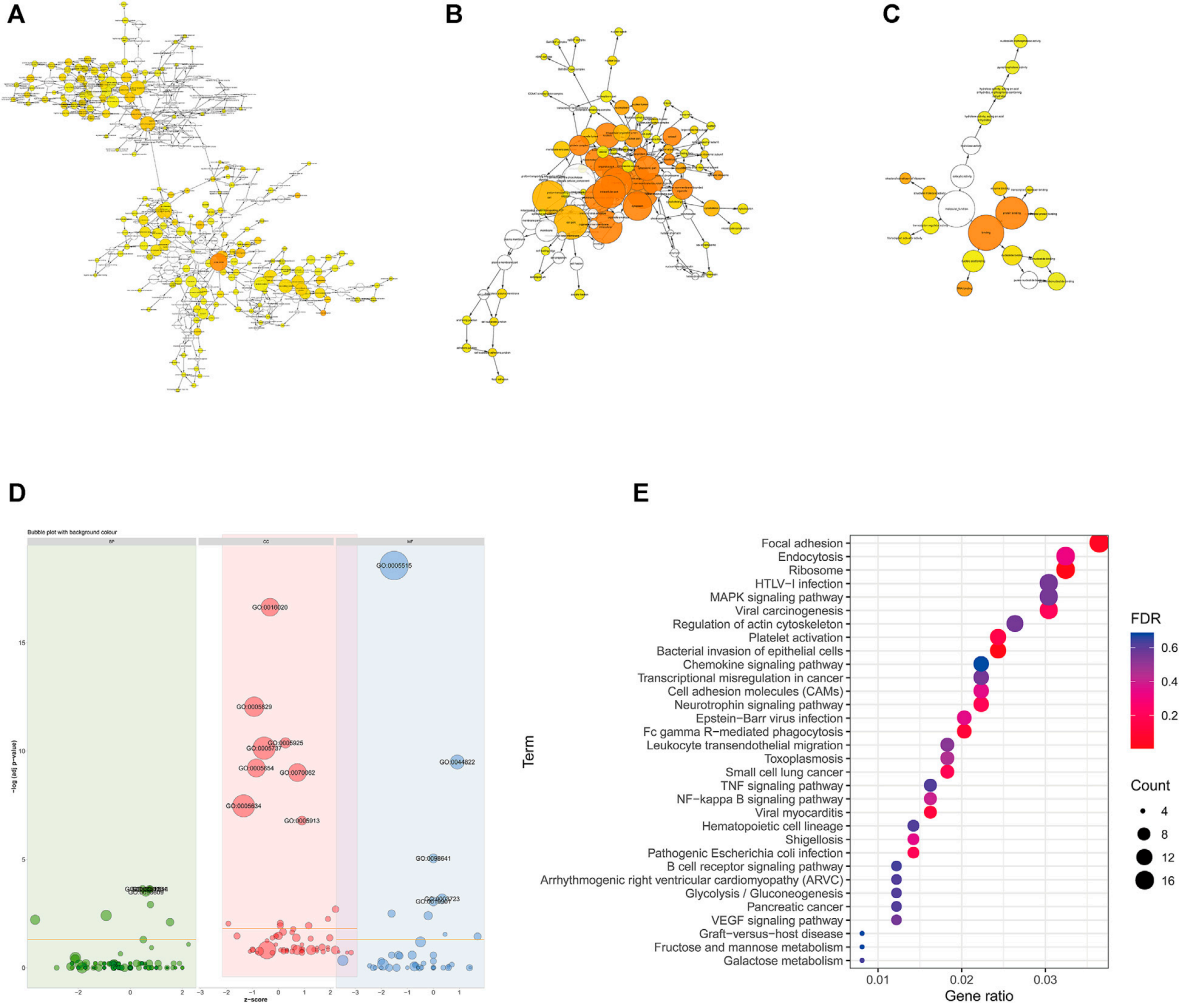
Functional enrichment of differentially expressed mRNAs. **(A–C)** Directed acyclic graph of GO analysis: BP **(A)**, CC **(B)** and MF **(C)**. **(D)** Bubble graph of GO analysis. Green represents BP, red represents CC, and blue represents MF. **(E)** KEGG pathway enrichment analysis. The color of the dot corresponds to the *p-value* range, and the size of the dot indicates the number of genes in the pathway.

The main biological functions of DE mRNAs were investigated through enrichment analysis of GO and KEGG pathways to further verify the possibility of pathophysiological processes. The biological process (BP), molecular function (MF) and cellular component (CC) categories associated with the DE mRNAs were displayed through a directed acyclic graph (DAG, Figures 3A–D). GO analyses indicated that the pathological processes involved in LAA ischemic stroke may regulated by DE mRNAs from different angles, such as cell-cell adhesion (GO:0098609), positive regulation of apoptotic process (GO:0043065), positive regulation of cell migration (GO:0030335), and megakaryocyte differentiation (GO:0030219). We also performed KEGG pathway analysis (Figure 3E), and the results showed that DE mRNAs were mainly related to cellular senescence (hsa04218), fluid shear stress and atherosclerosis (hsa05418), platelet activation (hsa04611), complement and coagulation cascades (hsa04610) and so on (Online Supplementary Tables S4, S5).

### Construction of the Protein-Protein Interaction Network

**FIGURE 4:**
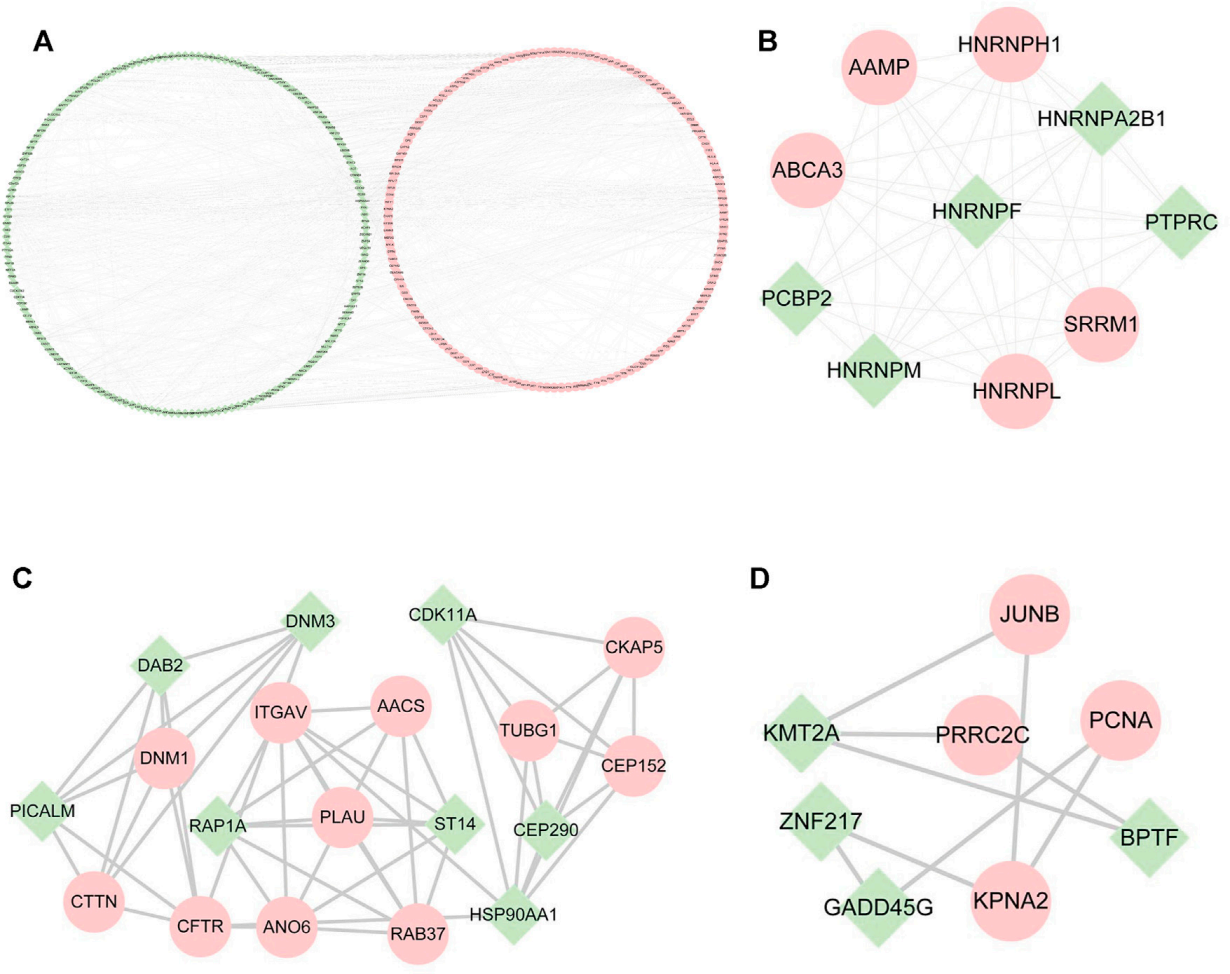
PPI networks among differentially expressed mRNAs and submodules. **(A)** PPI networks of all the DE mRNAs. **(B–D)** Submodules in PPI networks. Green represents downregulated mRNAs; pink represents upregulated mRNAs.

Through PPI analysis, a network of 522 nodes and 674 edges was generated from the DE mRNAs (Figure 4A). The Cytoscape plugin MCODE was used to extract 3 submodules from the PPI network (Figures 4B–D).

1) Module1 (score = 8.667) contains 10 nodes and 40 edges, among which heterogeneous nuclear ribonucleoproteins, such as HNRNPH1, HNRNPA2B1, HNRNPF, HNRNPM, and HNRNPL (Figure 4B), were the most abundant.
2) Module 2 (score = 5.889) contains 19 nodes and 53 edges and includes AACS, CKAP5, CDK11A, and DNM3 (Figure 4C).
3) Module 3 (score = 2.571) contains 8 nodes and 9 edges and includes JUNB, BPTF, PCNA, PRRC2C, and BPTF (Figure 4D).

### Construction of lncRNA-Related Molecular Networks

**FIGURE 5:**
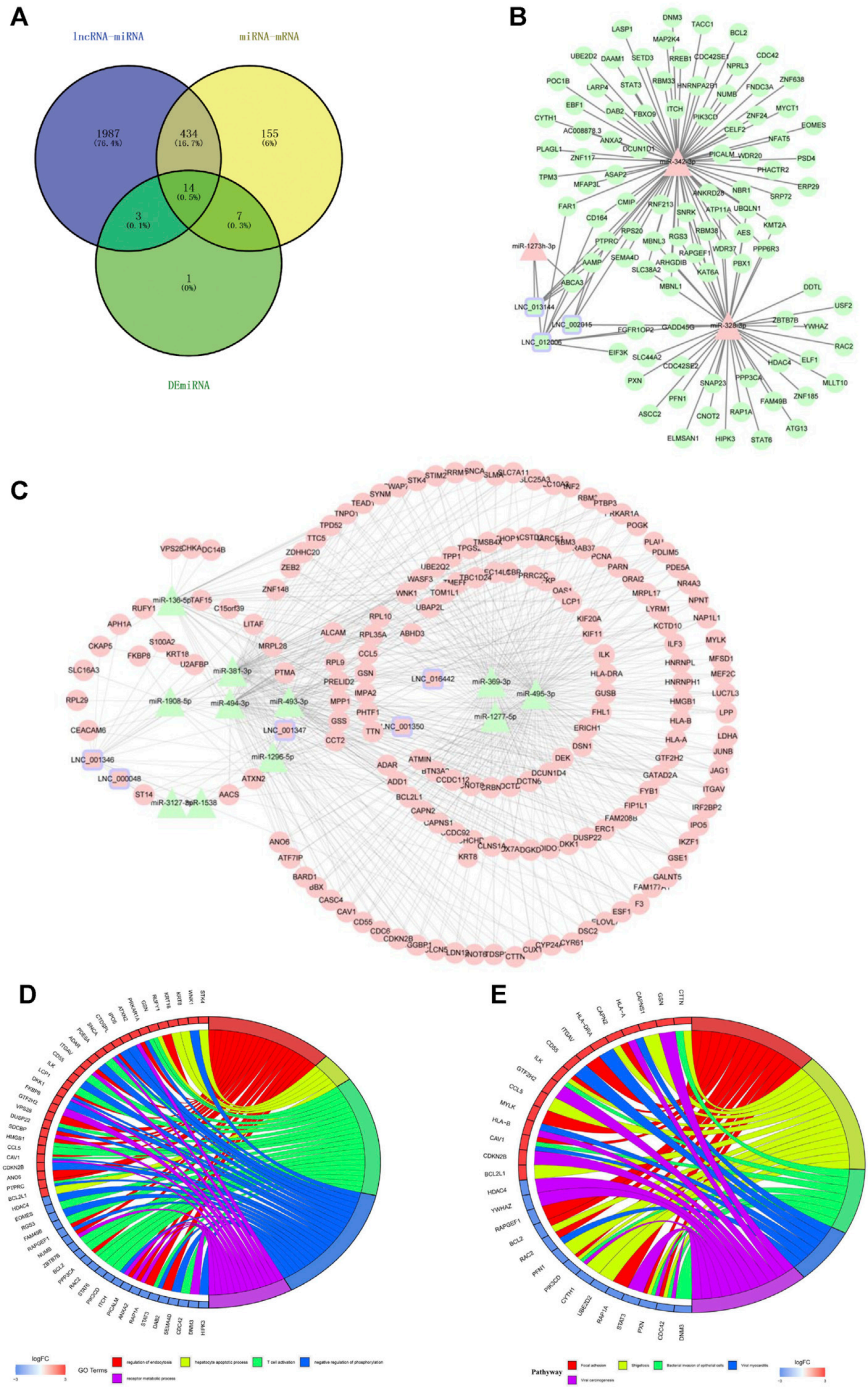
The lncRNAs-miRNAs-mRNAs networks and functional analysis. **(A)** The interaction of DE lncRNAs-miRNAs pairs, miRNAs-DE mRNAs pairs and DE miRNAs. **(B)** downregulated lncRNAs-upregulated miRNAs-downregulated mRNAs networks. **(C)** upregulated lncRNAs-downregulated miRNAs-upregulated mRNAs networks. Triangles represent miRNAs, circles represent mRNAs, squares with purple edges represent lncRNAs, green represents downregulated RNAs, and pink represents upregulated RNAs. **(D)** GO analysis of mRNAs in the networks. **(E)** KEGG analysis of mRNAs in the networks. Red represents upregulated mRNAs, and blue represents downregulated mRNAs.

According to the ceRNA hypothesis, lncRNAs could influence the expression or stability of the target mRNA by binding miRNA. In this study, we selected eight hub lncRNAs, which function related to AS and had higher fold change as well as had reads in each sample (lnc_000048, lnc_001346, lnc_001347, lnc_001350, lnc_002015, lnc_012006, lnc_013144, lnc_016442), to construct lncRNA-related networks. A total of 7274 DE lncRNAs-miRNAs pairs and 55,743 miRNAs-DE mRNAs pairs were screened out. Through the interaction of DE lncRNAs-miRNAs, miRNAs-DE mRNAs and DE miRNAs, 14 hub DE miRNAs (hsa-miR-1277, hsa-miR-1273h, hsa-miR-1296, hsa-miR-136, hsa-miR-1538, hsa-miR-1908, hsa-miR-3127, hsa-miR-328, hsa-miR-342, hsa-miR-369, hsa-miR-381, hsa-miR-493, hsa-miR-494, hsa-miR-495) were obtained (Figure 5A).

Figure 5B reveals a downregulated DE lncRNAs–upregulated DE miRNAs-downregulated DE mRNAs regulation pattern; conversely, Figure 5C reveals the upregulated DE lncRNAs-downregulated DE miRNAs-upregulated DE mRNAs pattern.

Functional enrichment was performed to understand the biological processes of the lncRNA-related networks. The top five GO and KEGG terms were visualized in R package (Figures 5D,E). The results showed that the target genes of ceRNA networks were related to immunity, endothelial inflammation, adhesion and apoptosis. All the results confirmed lncRNAs as powerful regulatory through ceRNAs mechnism in LAA stroke.

### Subcellular Localization of lncRNAs

**FIGURE 6:**
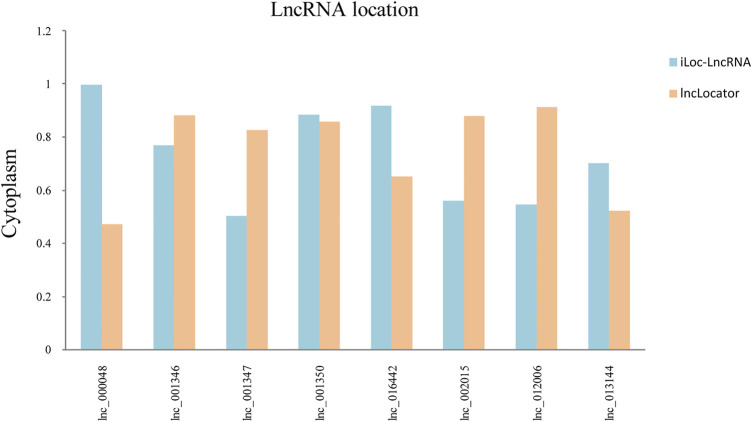
Subcellular localization of selected lncRNAs. Blue represents the iLoc-LncRNA database, and orange represents the lncLocator database.

The subcellular localization of lncRNAs determines their different regulatory modes. LncRNAs can sponge their interacting miRNAs to further regulate the stability and translation of an mRNA. The positions of eight hub lncRNAs were predicted by the lncLocator and the iLoc-LncRNA website (Figure 6). Lnc_002015 and lnc_001350 are most likely to be located in the cytoplasm, which suggests that they might function through ceRNA networks.

### Validation of the Differentially Expressed RNAs

**FIGURE 7:**
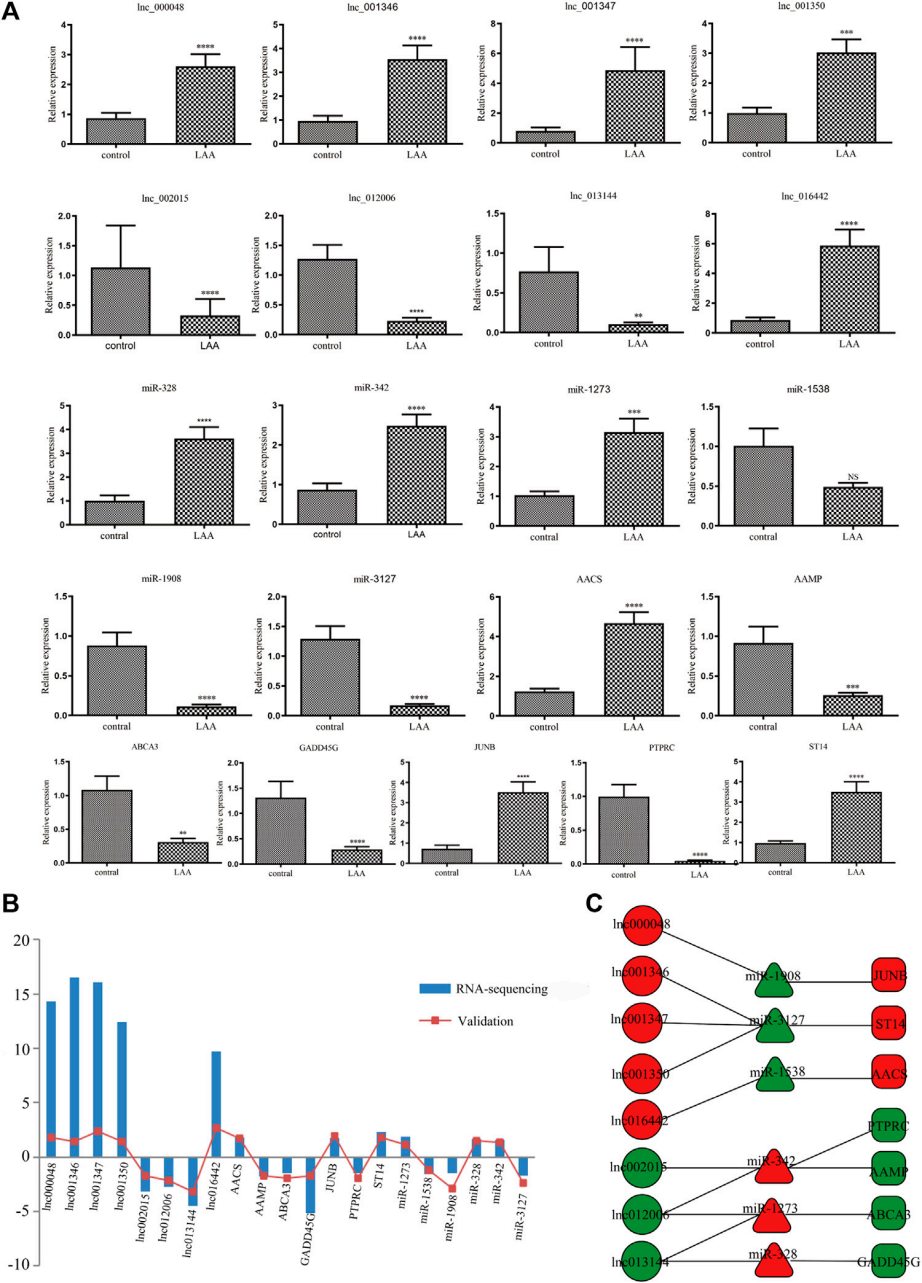
Validation of the expression of DE hub RNAs by qRT-PCR. **(A)** qRT-PCR was used to evaluate the expression of 21 DE hub RNAs in plasma-derived exosomes from patients with LAA stroke (n = 30) and healthy controls (n = 30). All data are shown as means ± SD. **(B)** The trend of expression between RNA-seq and qRT-PCR was consistent. **(C)** The networks of 21 hub exo-RNAs.

We extracted 8 lncRNA-related subnetworks for subsequent analysis. The criteria for selection were as follows: coexpression of lncRNAs and mRNAs; GO and KEGG analysis of mRNAs involved in atherosclerosis. A total of 21 hub genes of networks were selected (Figure 7C). To verify our findings, we confirmed the relative expression level of 21 hub genes in the validation set (LAA: control = 30:30) by qRT-PCR (Figure 7A). The results showed that the selected genes were differentially expressed between the LAA and control groups, which were consistent with our sequencing results (Figure 7B).

### Assessment of the Diagnostic Performance of the Selected exo-lncRNAs

**FIGURE 8:**
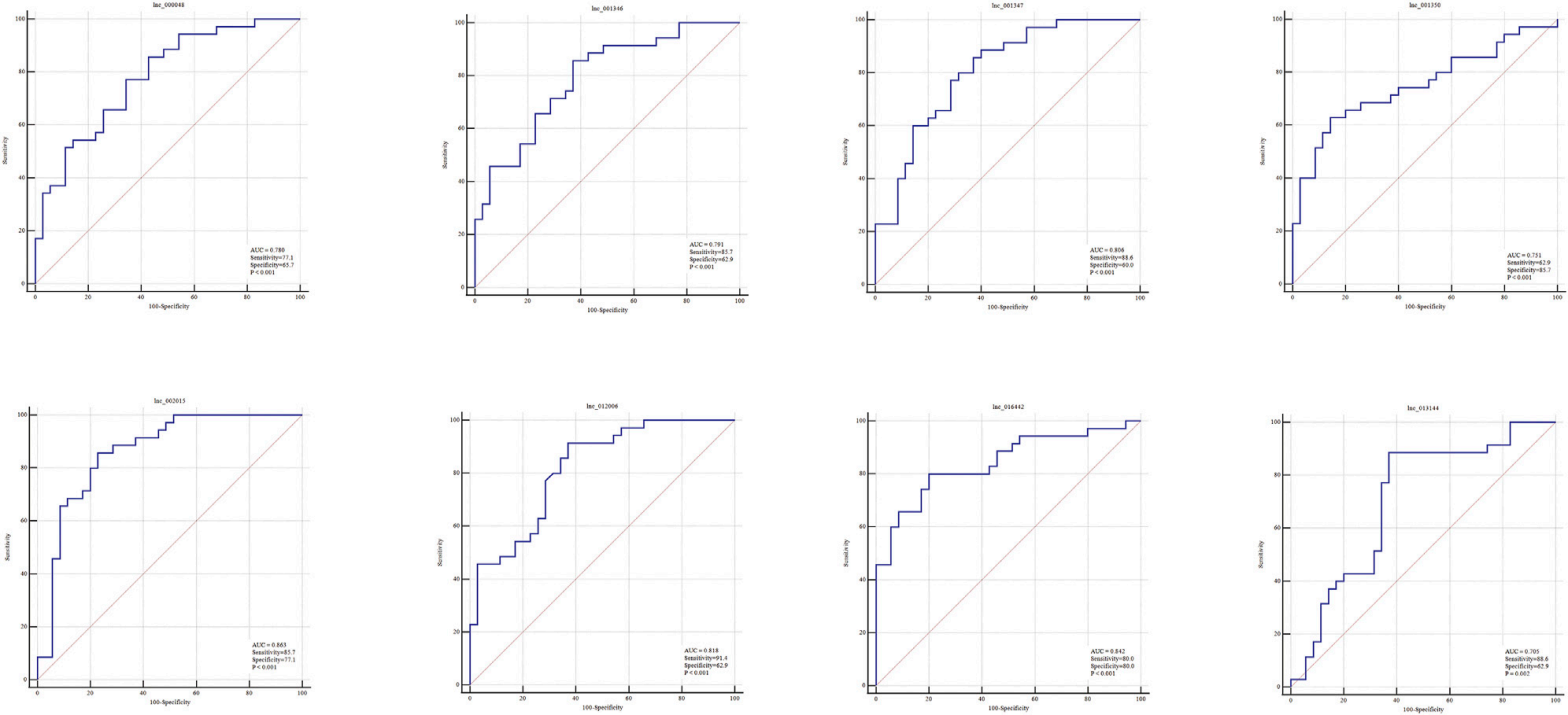
ROC curves (validation sample set) of 8 DE hub lncRNAs in plasma-derived exosome of LAA.

To evaluate the diagnostic performance of the selected lncRNAs for LAA stroke, we generated ROC curves in the validation set. The diagnostic performance of lnc_000048, lnc_001346, lnc_001347, lnc_001350, lnc_002015, lnc_012006, lnc_013144, and 016442, measured by the area under the curve (AUC), were 0.78, 0.791, 0.806, 0.751, 0.863, 0.818, 0.842, and 0.705, respectively (Figure 8).

### Correlation Between the 21 hub RNAs and Clinical Characteristics

**FIGURE 9:**
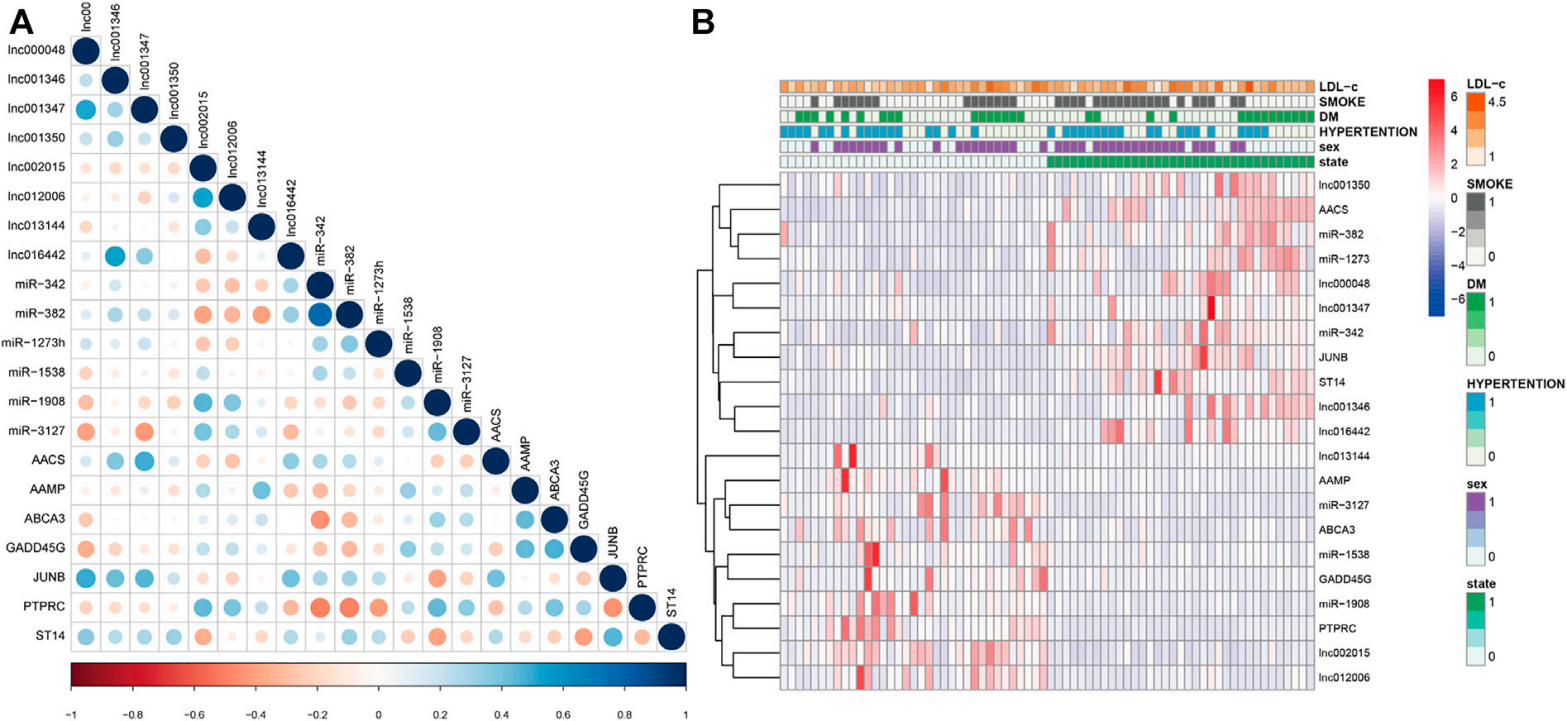
Correlation of selected exosomal hub RNAs and combination with clinical outcomes of LAA stroke patients. **(A)** The correlation of 21 exo-hub genes is shown by a circle map. Blue represents a positive correlation, red represents a negative correlation, and the depth of color and the size of dot correspond to the correlation coefficient. **(B)** Correlation of selected exoRNAs and clinical outcoms in LAA stroke patients. Red represents risk factors, and blue represents protective factors for LAA.

We analyzed the correlation between the 21 exo-hub genes. The results showed that the correlations of the 21 exo-hub genes were consistent with our predicted outcomes. As shown in Figure 9A, selected upregulated lncRNAs were negatively correlated with miRNAs (hsa-miR-342, hsa-miR-1273, hsa-miR-328) and coexpressed with mRNAs (JUNB, AACS, ST14). Furthermore, the analysis in combination with clinical outcomes demonstrated that upregulated exo-hub RNAs acted as risk factors for LAA stroke (Figure 9B).

### Exploration of the exo-lncRNA-related Diagnostic Model of LAA Stroke

**FIGURE 10:**
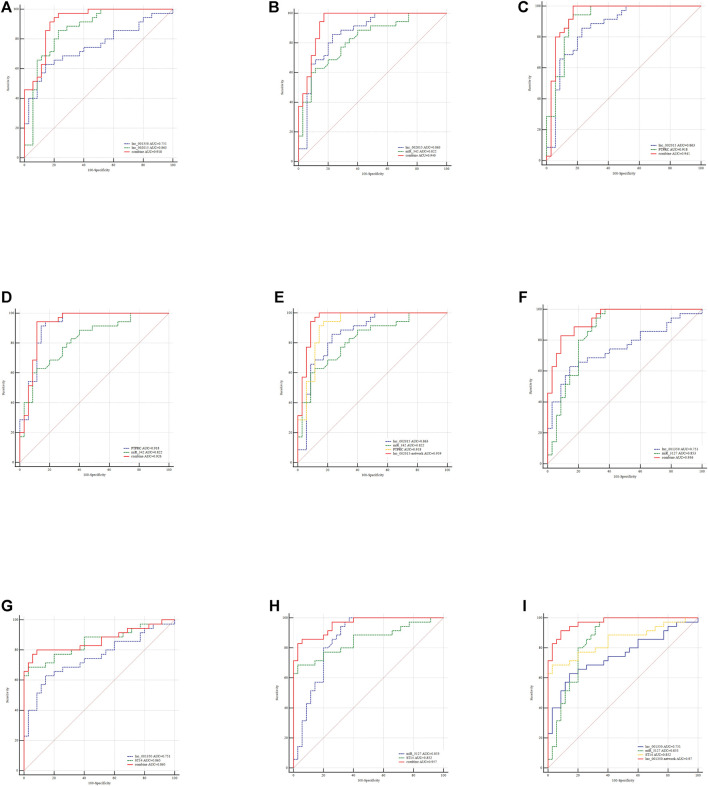
Evaluation of the diagnostic performance of the exo-lncRNA diagnostic panel and exo-lncRNA-related networks for LAA stroke. ROC analysis was used to evaluate the performance of the exo-lncRNA diagnostic panel **(A)**, lnc002015+miR-342 **(B)**, lnc002015+PTPRC **(C)**, miR-342+ PTPRC **(D)**, exo-lnc002015-related network **(E)**, lnc001350+miR-3127 **(F)**, lnc001350 + ST14 **(G)**, miR-3127 + ST14 **(H)**, and exo-lnc001350-related network **(I)**.

Combining risk factors may improve their diagnostic accuracy. Therefore, logistic regression analysis was performed to establish a diagnostic panel of the hub exosomal lncRNAs (lnc_000048, lnc_001346, lnc_001347, lnc_001350, lnc_002015, lnc_012006, lnc_013144, lnc_016442). The results showed that lnc_001350 (OR: 2.038, *p* < 0.05) promoted the onset of LAA stroke, whereas lnc_002015 (OR: 0.017, *p* < 0.005) was a protective factor. The probability of being diagnosed with LAA stroke was calculated using the following equation: Logit (P) = 1.127 + 0.712 × lnc_001350-4.07 × lnc_002015. The AUC of the predicted diagnostic panel was 0.918 (95% CI = 0.827–0.970, Figure 10A). However, the difference in AUC between lnc_002015 (0.863) and the panel (0.918) was not statistically significant (*p* = 0.085).

LncRNAs could be integrated with mRNAs and miRNAs as possible biomarkers for disease diagnosis. Furtherly, the exo-lncRNA (lnc_002015 and lnc_001350), which belongs to the factors of Logistic regresson diagnostic panel, related networks were analyzed by ROC analysis. The results showed that the AUCs of the lnc_002015- related network and lnc_001350- related network were 0.959 (95% CI = 0.883–0.992, Figure 10E, 0.970) (95% CI = 0.898–0.996, Figure 10I), respectively. Although combining two compositions of the network randomly could improve the diagnostic performance (Figures 10B–D,F–H), the network showed an obvious advantage over each combination in identifying LAA stroke patients. The differences between the AUCs of the network and each factor in the network were statistically significant (*p* < 0.05), which indicated that the exo-lncRNA-related networks might have higher diagnostic efficacy.

## Discussion

This is the first study to comprehensively analyze circulating exosomal lncRNA sequencing in LAA stroke and control. We determined the differential expression profile of exosomal lncRNAs and built lncRNA-related networks. The results showed that lncRNA-related networks exhibited better diagnostic performance than single factor, and these networks might be considered promising diagnostic models and provide novel insights into the mechanism in LAA stroke.

Exosomes protect lncRNAs from RNase-induced damage to maintain the concentration of lncRNAs in the blood (Guo et al., 2020; Kim et al., 2020), and the stability of exosomal lncRNAs makes them good potential diagnostic biomarkers. Hu et al. reported that the expression level of plasma exosomal lncRNAs was significantly upregulated in colorectal cancer patients, serving as biomarkers for the early detection of CRC (Hu et al., 2018). Kenji Takahashi et al. highlighted that highly upregulated circulating exosome-encapsulated lncRNAs were potential biomarkers of pancreatic cancer (Takahashi et al., 2020). Our current findings showed that exo-lncRNAs were significantly differentially expressed in our discovery samples. Furthermore, the results were verified by validation samples, which suggested that these exo-lncRNAs might have diagnostic potential in LAA stroke.

Recently, lncRNAs have been studied because they can regulate mRNA expression in many forms, including through competitive binding with miRNAs (Cesana et al., 2011). Through their interactions with the seed regions of miRNAs, lncRNAs can block an entire family of related miRNAs. This leads to the disinhibition of downstream target genes, ensuring the stability of the mRNA function (Huang et al., 2019). Le Qu et al. found that lncARSR could promote c-MET and AXL expression by acting as a ceRNA for miR-449 and miR-34 in renal cancer. Similarly, our study found that exo-lncRNA could bind one or more miRNAs and regulate multiple mRNAs, which was consistent with previous research.

Furthermore, functional enrichment (GO and KEGG pathway) analysis was performed on the target genes (mRNAs) of lncRNA-related networks. The results showed that lncRNA-related networks were involved in inflammation, cell adhesion, immunity and other functions. For instance, GO: 0050839, representing cell adhesion, has been proven to be related to atherosclerosis. Chadjichristos CE found that Cx40 promotes leukocyte adhesion to the endothelium and accelerates atherosclerosis (Chadjichristos et al., 2010). GO: 0006979, representing response to oxidative stress, also participates in atherosclerosis.Chen Y et al. revealed that fatty acid metabolism is disordered in response to oxidative stress from the mitochondria, which drives chronic inflammation and accelerates the process of atherosclerosis (Chen et al., 2015). Therefore, we speculated that the DE lncRNAs might regulate the progression of LAA stroke by targeting genes.

According to the functional annotation results, we selected eight lncRNAs that might be related to atherosclerosis. Based on ceRNA theory, we constructed eight lncRNA-related networks, such as lnc_002015/hsa-mir-342/PTPRC/AAMP and lnc001350/hsa-mir-3127/ST14. PTPRC, protein tyrosine phosphatase receptor type C, is a signaling molecule that regulates a variety of biological processes, including cell growth, differentiation and mitosis (Bottero et al., 2018). Recently, Pan et al. (2020) found that PTPRC was associated with atherosclerosis as a hub gene in the regulation of cell cycle progression. MiR-342 directly targets Akt1 through its 3′-UTR region and induces the downregulation of proinflammatory factors such as Nos2 and IL-6. Thus, miR-342 promotes the progression of atherosclerosis in Apoe/mice (Wei et al., 2013). The above two genes are related to the progression of atherosclerosis, implying that lnc_002015, as an important member of the ceRNA mechanism, has potential regulatory capacity in LAA stroke. This is the first study to imply that lnc_002015 might act as a molecular sponge of miR-342 to regulate the progression of atherosclerosis. More experiments are needed to explore the pathways of genes and the functions of this network.

Importantly, the logistic regression model revealed that lnc_001350 was positively associated with the risk of LAA stroke, whereas lnc_002015 was predicted as a protective factor. The diagnostic performance of lnc_002015 was consistent with the functional enrichment of the lnc_002015-related network. ROC curves revealed that all the selected exosomal hub genes had the potential to be diagnostic indicators.

To elevate the accuracy of diagnosis, we performed ROC analysis on the panel of the logistics equation and the corresponding exo-lncRNA related networks simultaneously and compared them. Interestingly, the AUCs all changed obviously when we integrated factors from both the regression equation and ceRNA networks into the analysis concurrently. However, there was no statistically significant difference between the diagnostic performances of the diagnostic panel and each factor in the panel. In contrast, exo-lnc_002015- and exo-lnc_001350-related networks showed improved diagnostic performance, which might due to the interactions among the genes. The results implied that the diagnostic possibilities of exo-lncRNA-related networks were higher than those of single factors in LAA stroke. Future validation of individual exo-lncRNA-related networks in a larger cohort is needed.

In conclusion, this study has clarified the differentially expressed exosomal lncRNAs in LAA stroke. More importantly, the results showed for the first time that the diagnostic performance of the exo-lncRNA-related network is superior to that of single factor, which might serve as a novel and accurate diagnostic method. Our findings may offer new perspectives to explore candidate targets for the diagnosis and mechanism of LAA stroke.

## Data Availability

The data presented in the study are deposited in the (GEO) repository, accession number (GSE173719).

## Abbreviations

LAA stroke, large artery atherosclerotic stroke; GEO, gene expression omnibus; DEGs, differentially expressed genes; PPI, protein–protein interactions; KEGG, Kyoto; encyclopedia of genes and genomes; Exo-IncRNA, exosomal IncRNA; ROC, receiver operating characteristic curve; AUC, area under curve.
