# Supplementary figures and images for: Novel Insights into the Potential Diagnostic Value of Circulating Exosomal IncRNA-Related Networks in Large Artery Atherosclerotic Stroke
Source: Front Mol Biosci. 2021 May 21;8:682769. doi: 10.3389/fmolb.2021.682769 (PMC8176956; doi:10.3389/fmolb.2021.682769)

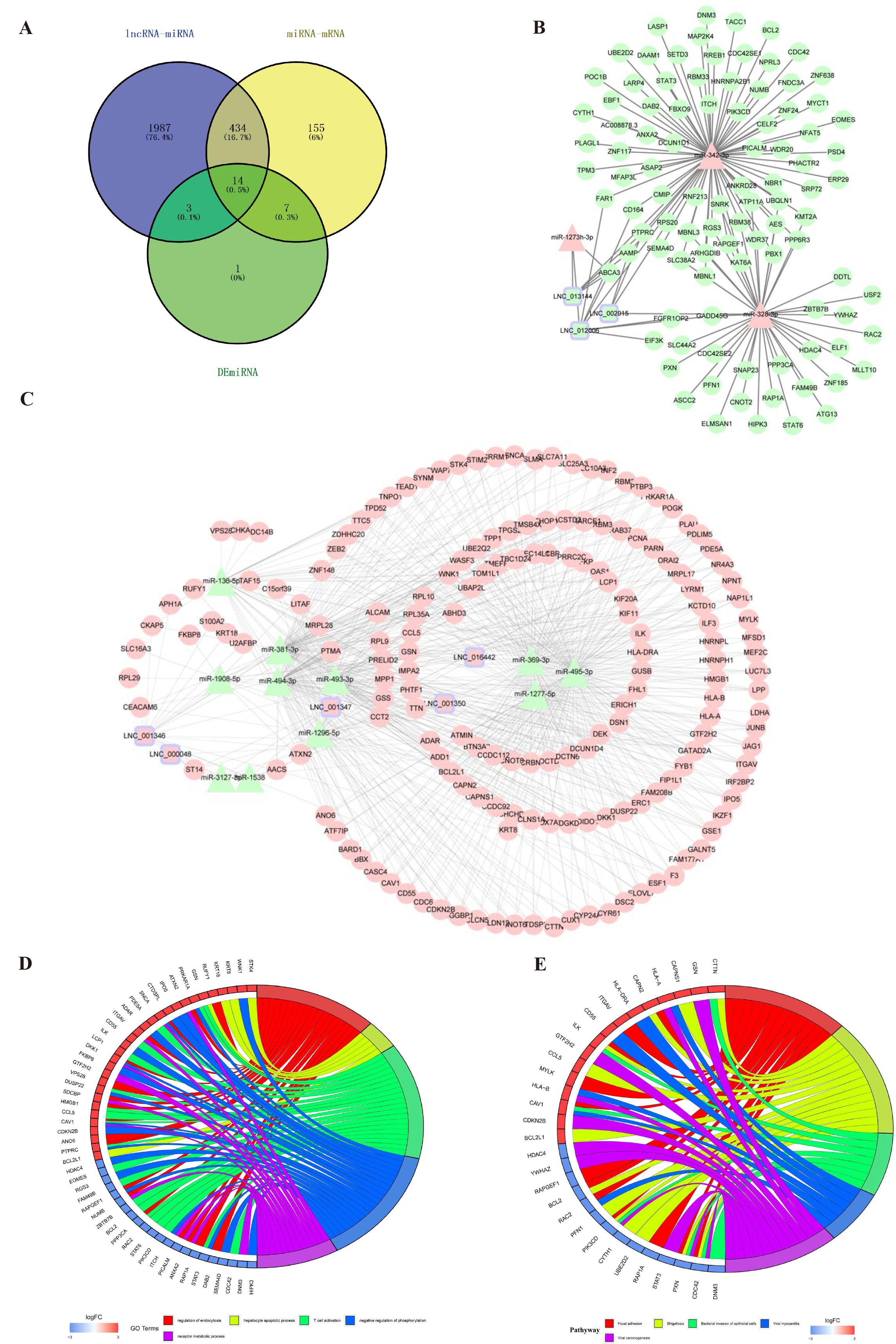

Supplement: Supplementary file 5 [file DataSheet1.ZIP › Figure 5.tif]

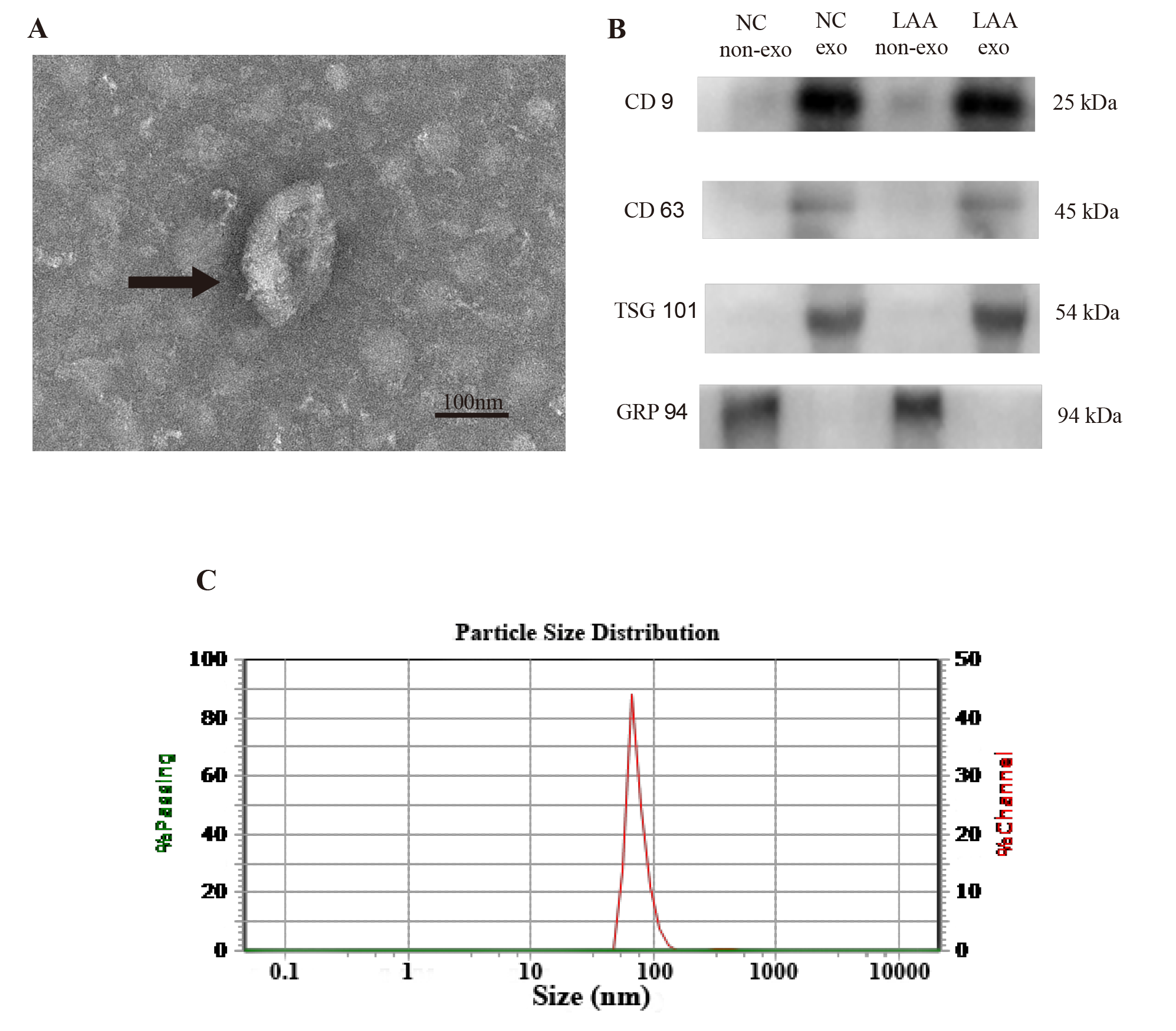

Supplement: Supplementary file 5 [file DataSheet1.ZIP › Figure 1.tif]

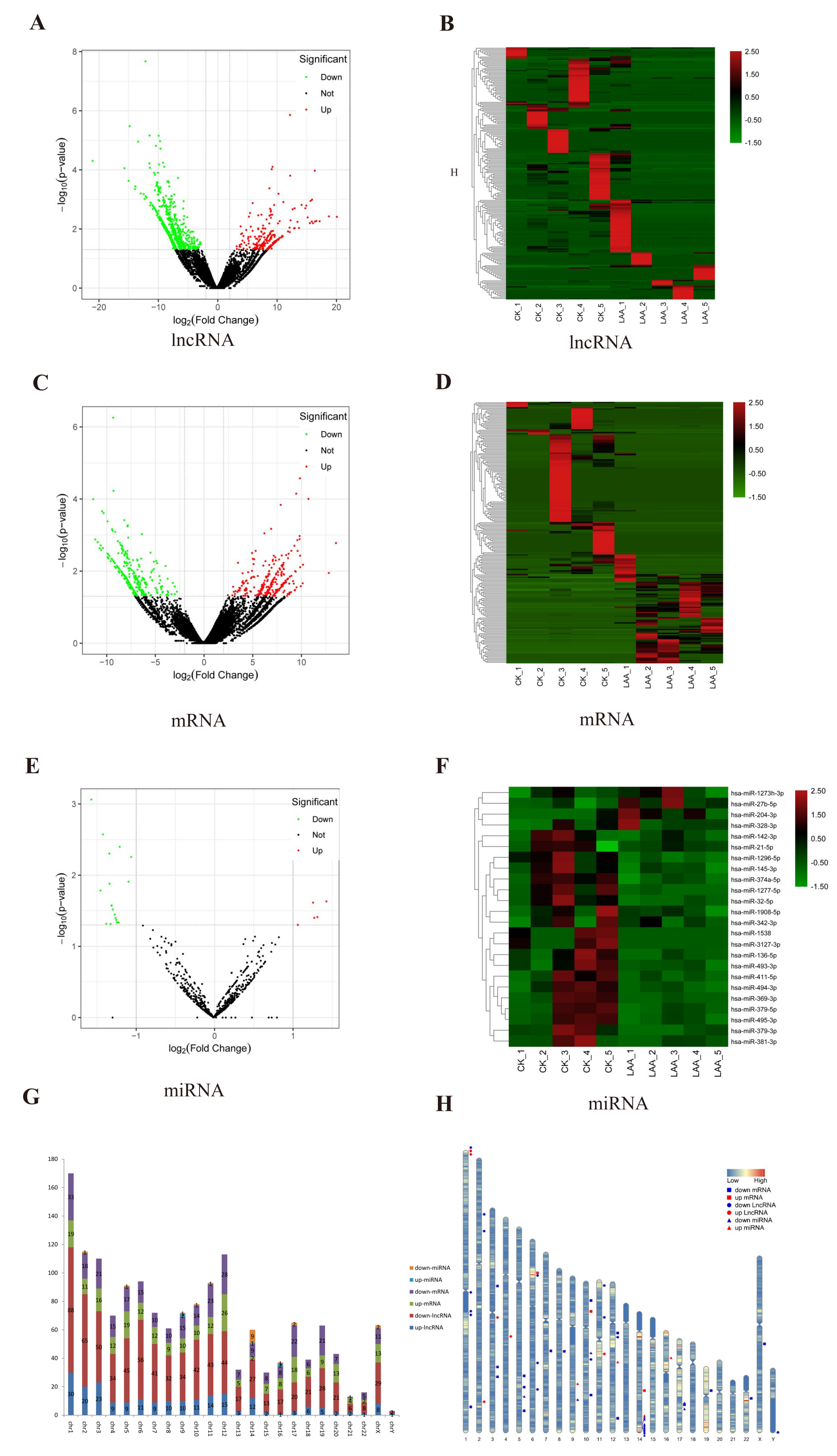

Supplement: Supplementary file 5 [file DataSheet1.ZIP › Figure 2.tif]

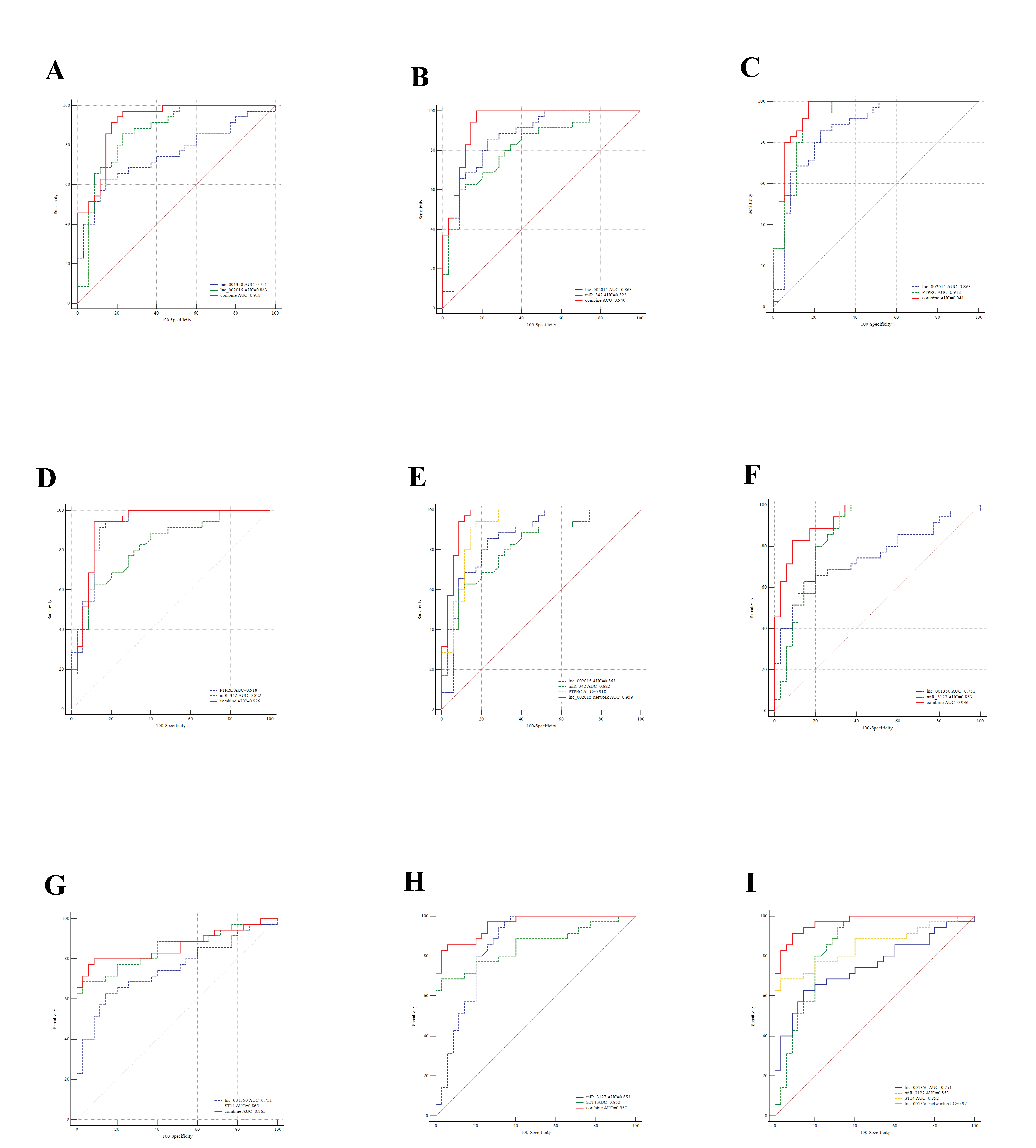

Supplement: Supplementary file 7 [file DataSheet2.ZIP › Figure 10.tif]

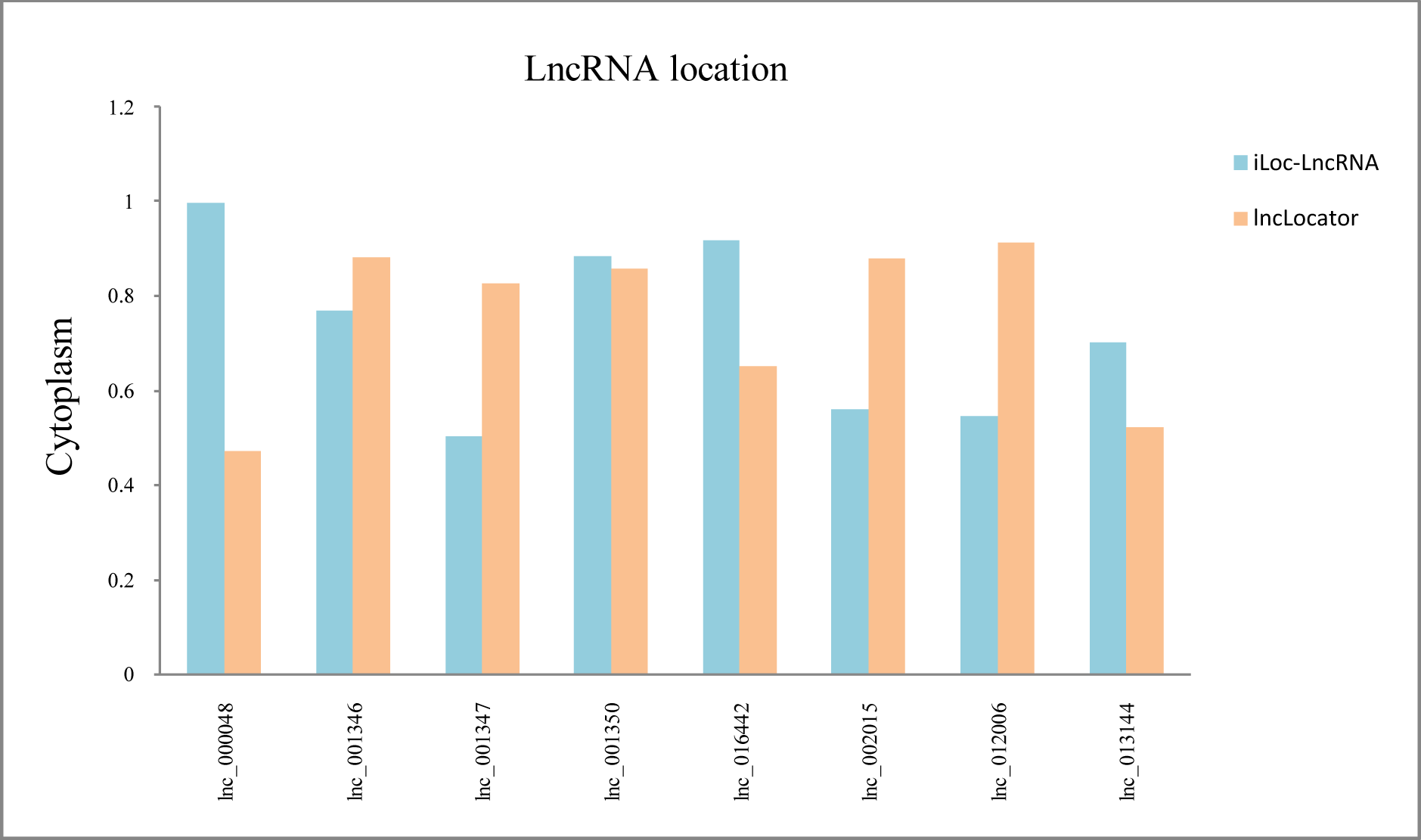

Supplement: Supplementary file 7 [file DataSheet2.ZIP › Figure 6.tif]

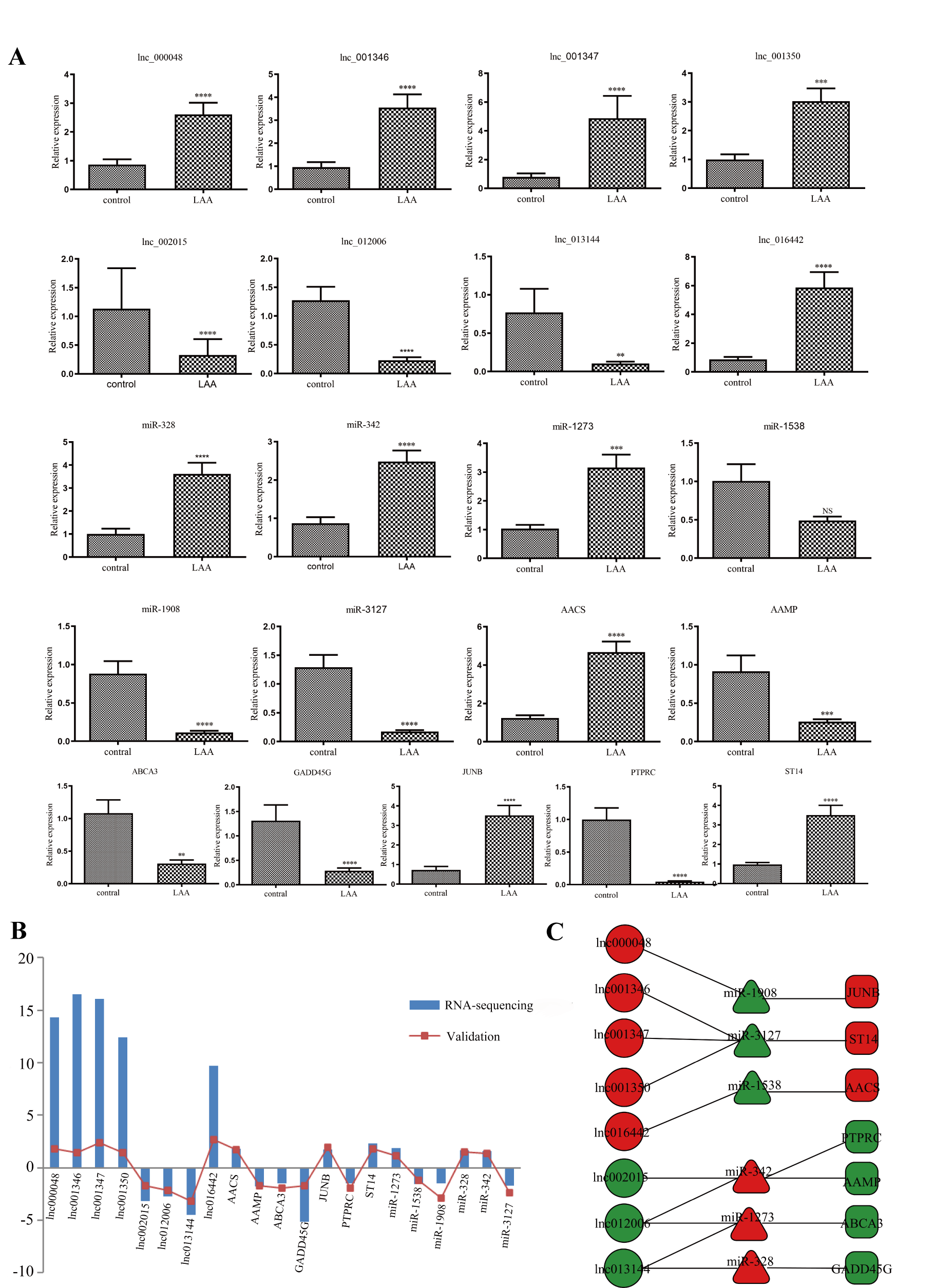

Supplement: Supplementary file 7 [file DataSheet2.ZIP › Figure 7.tif]

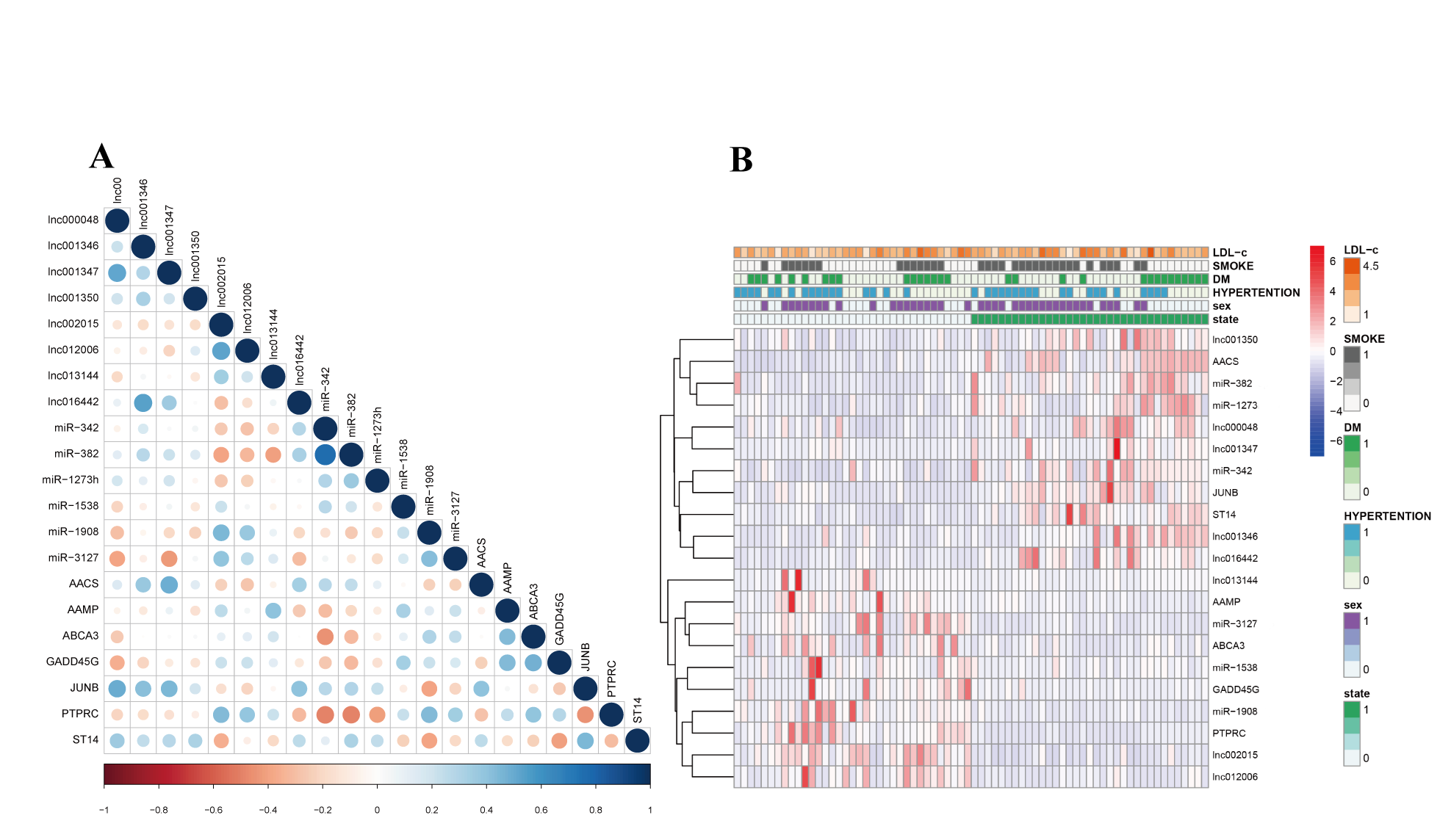

Supplement: Supplementary file 7 [file DataSheet2.ZIP › Figure 9.tif]
